# Functional organization and visual representations of human ventral lateral prefrontal cortex

**DOI:** 10.3389/fpsyg.2013.00371

**Published:** 2013-07-09

**Authors:** Annie W.-Y. Chan

**Affiliations:** Unit on Learning and Plasticity, Laboratory of Brain and Cognition, National Institutes of Health, National Institute of Mental HealthBethesda, MD, USA

**Keywords:** fMRI, prefrontal cortex, faces, eyes, functional organization

## Abstract

Recent neuroimaging studies in both human and non-human primates have identified face selective activation in the ventral lateral prefrontal cortex (VLPFC) even in the absence of working memory (WM) demands. Further, research has suggested that this face-selective response is largely driven by the presence of the eyes. However, the nature and origin of visual category responses in the VLPFC remain unclear. In a broader sense, how do these findings relate to our current understandings of lateral prefrontal cortex? What do these findings tell us about the underlying function and organization principles of the VLPFC? What is the future direction for investigating visual representations in this cortex? This review focuses on the function, topography, and circuitry of the VLPFC to enhance our understanding of the evolution and development of this cortex.

## Introduction

Faces, and particularly eyes, are some of the most salient visual and biological stimuli in the environment. In addition to extract information for identification, we look at others' faces and eyes to read their intentions and emotions in order to facilitate communication, especially when verbal information is not available. The neural representations of faces and eyes in the ventral temporal visual cortex and the banks of the superior temporal sulcus have been well explored in both humans (Kanwisher et al., 1997; Allison et al., 1999; McCarthy et al., 1999; Puce et al., 1999; Haxby et al., 2000a,b; Hoffman and Haxby, 2000; Tong et al., 2000; Haxby et al., 2002; Nummenmaa et al., 2010; Carlin et al., 2011, 2012) and monkeys (Perrett et al., 1982, 1984, 1985; Desimone et al., 1984; Tsao et al., 2003; Pinsk et al., 2005). In contrast, representation of such stimuli in the lateral frontal cortex has received relatively less attention despite evidence highlighting face-selective responses in the ventral lateral prefrontal cortex (VLPFC) in both human and non-human primates even in the absence of **working memory** (WM) demands (Wilson and Goldman-Rakic, 1994; O'Scalaidhe et al., 1997; Scalaidhe et al., 1999; Tsao et al., 2008a,b; Rajimehr et al., 2009; Chan and Downing, 2011). The nature and origin of such activation in the VLPFC remain unclear. This review will address what these findings reveal about the underlying function and organization of the lateral prefrontal cortex.

To date, much research has suggested that lateral prefrontal cortex is a site of convergence of information (Macko et al., 1982; Goldman-Rakic, 1987, 1996a,b; Wilson et al., 1993; Rao et al., 1997; Rainer et al., 1998; Romanski, 2004) from the dorsal (or “where”) and ventral (or “what”) pathways (Ungerleider and Mishkin, 1982; Miler and Goodale, 1995; Kravitz et al., 2011, 2013). The lateral prefrontal cortex is thought to have a central executive role in regulating and maintaining information “on line” by retrieving relevant long-term representation from these two pathways (Shallice, 1982; Baddeley et al., 1986; Goldman-Rakic, 1987). It is therefore thought that the organization of the lateral prefrontal cortex is based on the specific parietal-frontal and occipitotemporal-frontal connections. Further, the neural responses in the lateral prefrontal cortex have been argued to reflect the biases to these visuospatial and object-featural inputs (Fuster and Alexander, 1971; Jacobson and Trojanowski, 1977; Markowitsch et al., 1985; Goldman-Rakic, 1987). Beyond the “what vs. where” distinction, recent research has also proposed other organizational principles. For example, some evidence suggests a hierarchical functional organization of abstractness in the prefrontal cortex, from concrete to abstract representations, along a rostral-caudal gradient (Koechlin et al., 2003; Badre, 2008; Badre et al., 2009; Blumenfeld et al., 2012). Such organization has been found in both dorsal lateral prefrontal cortex (DLPFC) and VLPFC, with point-to-point functional connections to the dorsal medial prefrontal cortex (Kouneiher et al., 2009; Taren et al., 2011; Blumenfeld et al., 2012). In addition, a “Hot vs. Cold” distinction (Brown and Braver, 2005; Murray et al., 2007) has been suggested for medial and lateral frontal cortex, with the medial region focused on cognitive processing containing affective stimuli (“hot”), whilst the lateral region is more concerned with basic sensory motor stimuli (“cold”). It is worth noting that all of these organizational principles may not necessarily be orthogonal to each other (see O'Reilly, 2010), such that each of these principles may able to explain some variance in the activation pattern. Finally, one of the main controversies over the underlying organization of this cortex concerns whether the lateral prefrontal cortex is organized in terms of distinct functions/processes (**domain general**) or based on specific information/content (**domain specific**). Furthermore, one major problem confronted by researchers is that the role of the lateral prefrontal cortex has been examined using a variety of tasks and stimuli, making it very difficult to determine the necessary factors contributing to the organizational of the lateral prefrontal cortex.

Given that a great deal of research (Petrides and Milner, 1982; Goldman-Rakic, 1987; Courtney et al., 1996, 1997, 1998; Duncan and Owen, 2000; Haxby et al., 2000b; Miller, 2000; Romanski, 2004) of the prefrontal cortex revolves around the notion of domain general vs. domain specific, it is therefore crucial to elucidate the nature and origin of category-selective visual responses in the VLPFC, in order to expand our insight into the function and development of the prefrontal cortex.

This review not only aims to provide an overview of current evidence on visual representations in the VLPFC, but also aims to clarify the potential underlying organizational principles of this cortex. In the light of recent evidence from several research groups (Tsao et al., 2008a,b; Rajimehr et al., 2009; Chan and Downing, 2011), this review article will examine (1) neuroimaging evidence for visual face representation in the VLPFC from both human and non-human primates. (2) The extent to which VLPFC exhibits biases for parts of the visual field, which may be inherited from the high-level ventral visual cortex, (3) the functional organization of the VLPFC, (4) the connectivity between the lateral prefrontal cortex and other anatomically or functionally related brain regions, and finally (5) I will evaluate the nature and origin of the visual responses in the VLPFC cortex in the context of domain specific vs. domain general hypotheses.

## Visual face representation in ventral lateral prefrontal cortex

### Non-human primates

More than a decade ago, monkey physiology studies provided important findings to support the claim that visual category representations exist in the VLPFC. In particular, O'Scalaidhe et al. (1997); Scalaidhe et al. (1999) identified a small number of highly face-selective neurons near the inferior prefrontal convexity below the principal sulcus in monkeys. The responses of these face-selective neurons were equally robust for face stimuli in both WM task-trained monkeys and “naïve” monkeys who had not been trained on WM tasks. This distinctive population of neurons responded strongly to faces but weakly or not at all to non-face items such as common objects, scrambled faces, and simple colored shapes, supporting the claim that these neurons are category-selective. More recently, functional magnetic resonance (fMRI) studies in non-human primates (Tsao et al., 2008a,b; Rajimehr et al., 2009) have also reported visual category responses in the VLPFC. Specifically, these studies have all identified clusters in the inferior frontal cortex below the principal sulcus that responded highly selectively to images of faces, compared to other common objects, in the absence of WM demands.

### Human FMRI

The study of visual category representations in humans has largely focused on occipito-temporal cortex along the ventral visual pathway. For example, numerous brain-imaging studies have reported regions in the occipito-temporal cortex showing stronger responses to one particular category of stimuli compared to others. These include the face-selective Fusiform Face Area (FFA; Kanwisher et al., 1997; Allison et al., 1999; McCarthy et al., 1999; Puce et al., 1999), the body-selective Extrastriate Body Area (EBA; Downing, 2000; Chan et al., 2004; Downing et al., 2006; Taylor et al., 2007, 2010; Bracci et al., 2010; Chan et al., 2010; Chan and Baker, 2011; Ewbank et al., 2011) and Fusiform Body Area (FBA; Peelen and Downing, 2005; Taylor et al., 2007, 2010; Schwarzlose et al., 2008; Ewbank et al., 2011), the scene- and building- selective Parahippocampal Place Area (PPA; Epstein and Kanwisher, 1998; Walther et al., 2009; Kravitz et al., 2011; MacEvoy and Epstein, 2011; Harel et al., 2013), and the object-selective Lateral Occipital Complex or LOC (Grill-Spector et al., 2001; Downing et al., 2007). However, only a handful of recent studies have reported evidence for category-selective representations in the lateral prefrontal cortex, in particular for faces. For example, face-selective responses were reported in the VLPFC when contrasting 19 other object categories during passive viewing (Downing et al., 2006). Further, studies by the Tsao et al. (2008b) and Rajimehr et al. (2009) have also reported a face selective patch near the right inferior frontal sulcus in a number of human participants. Rajimehr et al. (2009) has further revealed that the face patch was located anterior and inferior to the frontal eye field (FEF).

**Figure 1:**
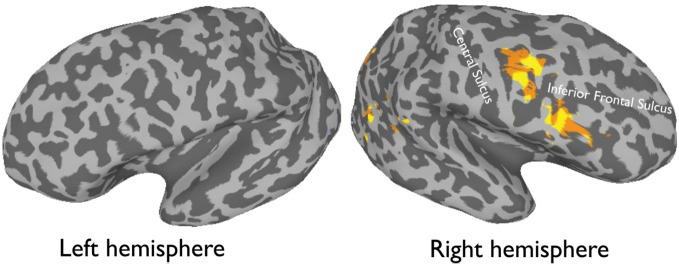
**An average activation map of 12 participants, 1-back task (faces > objects, random effect *p* < 0.001, *t* = 4.20) overlaid onto an inflated**.

**Figure 2:**
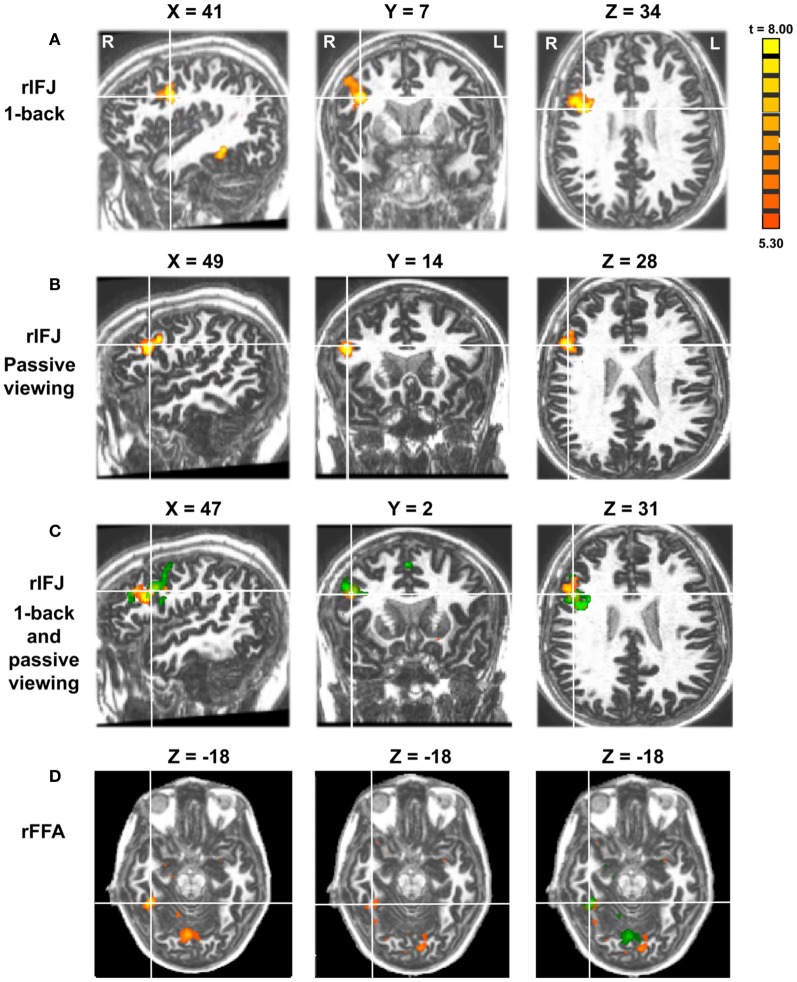
Panel **(A)** shows activations in the rIFJ and the rFFA during a 1-back task. Panel **(B)** shows activations in the rIFJ during passive viewing. Panel **(C)** shows the activation overlap in the rIFJ for both tasks (green for 1-back, yellow for passive viewing). Panel **(D)** shows the activation overlap in the FFA for both tasks (green for 1-back, yellow for passive viewing). All regions are defined by the contrast of faces-tools. (*p* < 0.0001, *t* = 5.30).

**Figure 3:**
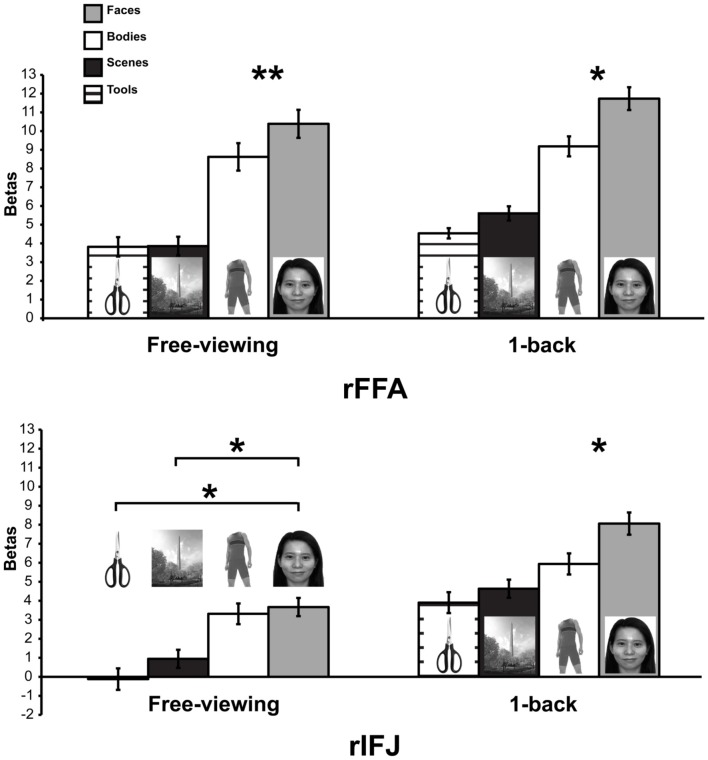
**Responses of rFFA and rIFJ, based on independent functional localizers, to faces, headless bodies, tools, and outdoor scenes, in both free-viewing and 1-back tasks**. Response magnitudes indicate beta weights from general linear models fit to the aggregate data from each region of interest. Error bars indicate standard error of the mean. Asterisks indicate significant differences between conditions: ^*^p < 0.05; ^**^p < 0.01.

**Figure 4:**
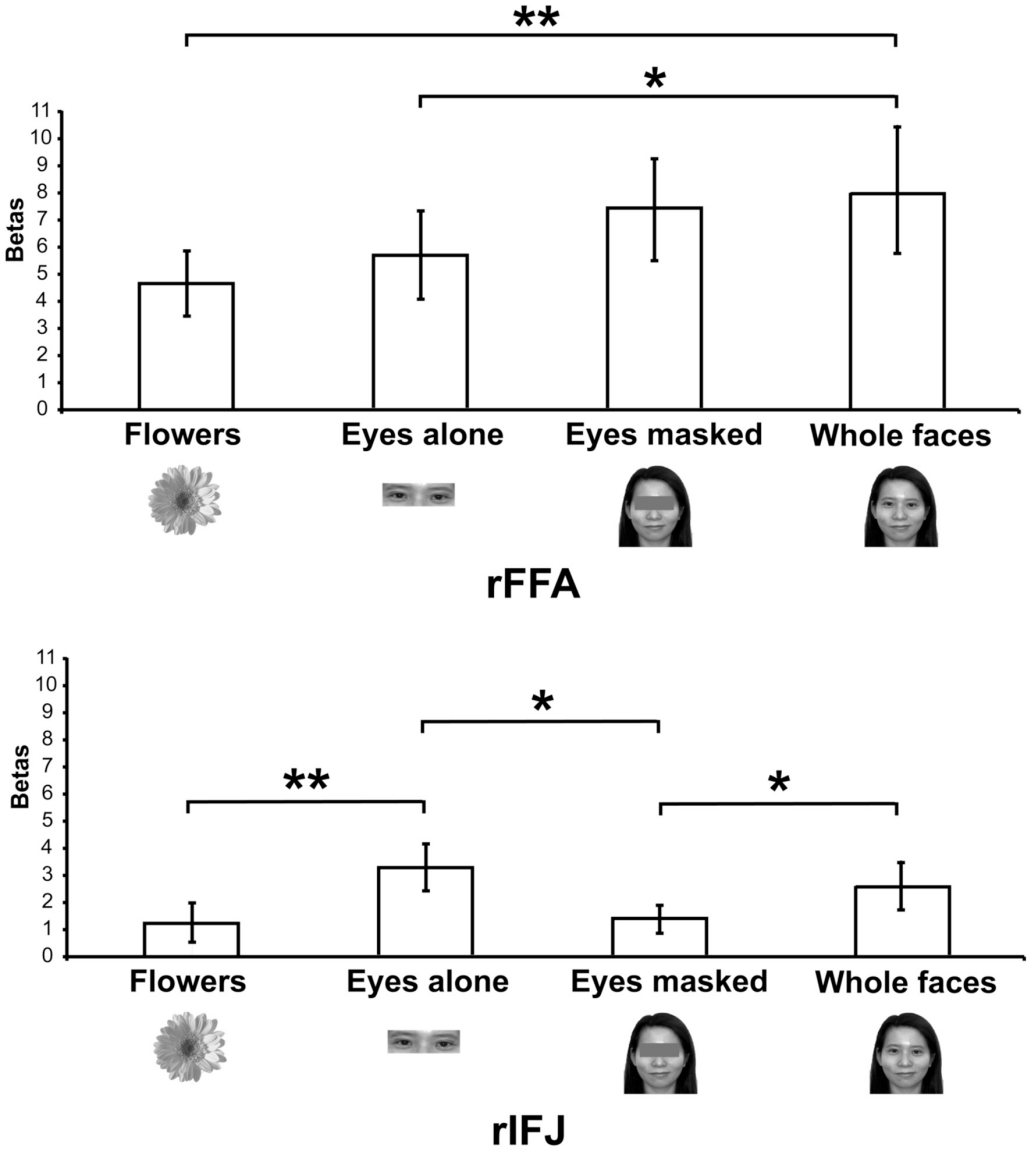
**Responses of rFFA and rIFJ, based on independent functional localizers, to flowers, whole faces, eyes, and faces with eyes masked**. Conventions as in Figure 2. Asterisks indicate significant differences between conditions: ^*^p < 0.05; ^**^p < 0.01.

More recently, (Chan and Downing, 2011) identified the specific location of this frontal face activation at the junction of pre-central sulcus and the inferior frontal sulcus or in the vicinity of the so-called right inferior frontal junction (rIFJ, Figure 1). This location is near to, but distinct from, areas associated with eye movement execution (such as the frontal eye fields or FEF). The activation lies approximately in the ventral inferior region of BA 9, neighboring the superior border of BA 44. It was found that these strong face responses in the rIFJ do not depend on WM demands. In particular, there were strong responses for both faces and body parts during passive viewing, and the responses for faces were stronger during a simple 1-back task (Figures 2, 3). Intriguingly, further investigation (see Figure 4) demonstrated that the rIFJ shows a strong preference for presentation of a pair of eyes in the absence of any other facial features. The eyes alone condition elicited the strongest responses relative to whole face, eyes masked (whole faces with eyes occluded), and the control condition, flowers. In contrast, the face-selective FFA in ventral temporal cortex responded significantly more weakly to eyes alone compared with the whole face and eyes masked conditions, and the response was not significantly different from that to flowers. Further, unlike IFJ, responses from the FFA were not driven by the presence of the eye, as activation for the whole face condition was indistinguishable from the eyes masked condition. In sum, these results suggest the VLPFC is driven by the presence of the eyes, not the face.

Evidence for a frontal face-selective response is not confined to the fMRI literature. For example, in evoked potential recordings (ERP), the vertex positive potential (VPP; Joyce and Rossion, 2005; Sadeh et al., 2011), recorded from the frontal channel, has been reported to show stronger amplitude for faces relative to objects, and its magnitude is comparable to the face selective N170 component in the temporal-occipital scalp (Rossion et al., 2003; Itier and Taylor, 2004; Bentin et al., 2006). The VPP is therefore thought to be another distinct face-selective component (Joyce and Rossion, 2005). In summary, converging evidence not only demonstrates that visual representation can be found in the lateral prefrontal cortex, but also suggests that the representation is domain specific, strongly biased to eyes and faces. In the following section, I will turn away from high-level visual properties and ask whether VLPFC shows lower level functional properties such as field biases that may be inherited from the ventral visual cortex.

## Visual field biases in ventral lateral prefrontal cortex

Effects of retinotopy are known to be present in both early and higher order visual areas. If face-selective responses in the VLPFC are mirroring the visual representations in the visual ventral pathway then, we may able to find some evidence of retinotopic biases in the VLPFC.

Early visual cortex is organized in terms of systematic retinotopy, with parts of cortex representing specific locations of visual input on retina, producing maps of visual space expressed in terms of eccentricity and polar angle (Sereno et al., 1995; DeYoe et al., 1996; Engel et al., 1997; Tootell et al., 1997; Wandell et al., 2007). Recent studies have demonstrated that retinotopic information extends even into high-level category-selective regions in **the ventral pathway** (Hasson et al., 2002; Brewer et al., 2005; Larsson and Heeger, 2006; Arcaro et al., 2009; Weiner and Grill-Spector, 2011). In particular, there appears to be an eccentricity bias along ventral temporal cortex with peripheral visual stimuli preferentially represented in medial regions of the ventral cortex, while foveal stimuli are preferentially represented in more lateral regions. This eccentricity bias may help explain the cortical locations of category-selective regions (Hasson et al., 2002; Malach et al., 2002; Levy et al., 2004). Tasks such as face recognition and reading require fine-grained visual analysis, and thus face- and visual word-selective regions are found in foveal biased cortex. In contrast, selective activation for stimuli that are typically experienced in peripheral vision (e.g., scenes) are found in peripherally-biased cortex.

Are such biases also present in lateral prefrontal cortex? Research in non-human primates has demonstrated that retinotopic biases also extend into the VLPFC. For example, near the face-selective cells reported by O'Scalaidhe et al. (1997); Scalaidhe et al. (1999), previous studies have reported neurons with foveal biases (Suzuki and Azuma, 1983). Furthermore, it has been reported (O'Scalaidhe et al., 1997; Scalaidhe et al., 1999) that a subset of face-selective cells in monkeys responded strongest to stimuli presented in the fovea, and weakly to stimuli presented in the periphery with weakest responses to peripheral presentations of spots of light. While peripherally presented faces only elicited very weak responses, these responses were still stronger than the responses to peripherally presented spots of light.

In humans, several studies (Hagler and Sereno, 2006; Saygin and Sereno, 2008) have reported polar angle mapping in the lateral prefrontal cortex and, in particular, regions ventral to FEF (near the IFJ). Further, it has been also shown that biological stimuli elicit a strong polar angle bias in the lateral prefrontal cortex. For example, Hagler and Sereno (2006) used phase-encoded face stimuli to found a visual field map for face stimuli in the lower, mid, as well as upper visual fields in the lateral prefrontal cortex near the IFJ. In addition, another study (Saygin and Sereno, 2008), using point-light biological stimuli, reported predominately mid to upper visual field responses while participants performing a 2-back WM task while maintaining fixation.

In sum, there is evidence in both human and non-human primates for spatial information with retinotopic biases emphasizing the fovea, similar to that reported in lateral regions of ventral temporal cortex. In the following section, I will discuss some of the evidence on visuospatial and visual-featural activations in the VLPFC and how these activations may able to give us clues regarding the underlying functional organization of this cortex.

## Functional organization in lateral prefrontal cortex

Research in the non-human primate has suggested that information from the ventral (“what”) and dorsal (“where”) pathways converges in the lateral prefrontal cortex in order to achieve complex goal-directed behavior. Specifically, it has been argued Macko et al., 1982; Goldman-Rakic, 1987; Wilson et al., 1993; Goldman-Rakic, 1996b; Rao et al., 1997; Rainer et al., 1998; Romanski, 2004 that outputs from **the dorsal pathway** extend into the DLPFC, primarily representing spatial information (also see Meyer et al., 2011). In contrast, outputs from the ventral pathway extend into the VLPFC, primarily representing object-featural information. This has led to the hypothesis that functional organization of the lateral prefrontal cortex is domain specific, receiving segregated information arising from the two pathways. Specifically, neurons in the DLPFC are tuned to spatial information and hence active during a spatial WM task (Rainer et al., 1998), while neurons in VLPFC are more tuned to object information and thus show robust activity when viewing face and object stimuli (Wilson et al., 1993; O'Scalaidhe et al., 1997; Scalaidhe et al., 1999; Tsao et al., 2008b; Rajimehr et al., 2009).

However, others have provided evidence against the domain specific hypothesis, arguing that the lateral prefrontal cortex is a domain general “multi-tasking” region, and is involved in a broad range of cognitive processes including selective attention, planning, WM, delayed matching, task-switching, inhibition, visual association, and visual categorization, using a variety types of stimulus, for example checker boards, color patches, simple shapes, patterns, letters, and other high-level visual stimuli (Rushworth et al., 1997; Asaad et al., 1998, 2000; Passingham et al., 2000; Freedman et al., 2001; Rossi et al., 2007, 2009).

Much of the debate on the functional organization of the VLPFC comes from research in visual WM in humans. Based on the idea that the object and spatial information converge in the lateral prefrontal cortex, previous work has aimed at identifying the neural substrates underlying object and spatial working memories (McCarthy et al., 1994; Courtney et al., 1996, 1997, 1998; Owen et al., 1996; Ungerleider et al., 1998; Postle and D'Esposito, 1999; Haxby et al., 2000b; Nystrom et al., 2000; Postle et al., 2000; Stern et al., 2000; Druzgal and D'Esposito, 2003; Sala et al., 2003; Volle et al., 2008). However, due to a lack of reproducibility across studies, the foci of activation for spatial and object WM as well as a clear double dissociation between DLPFC and VLPFC remain debatable.

**Figure 5:**
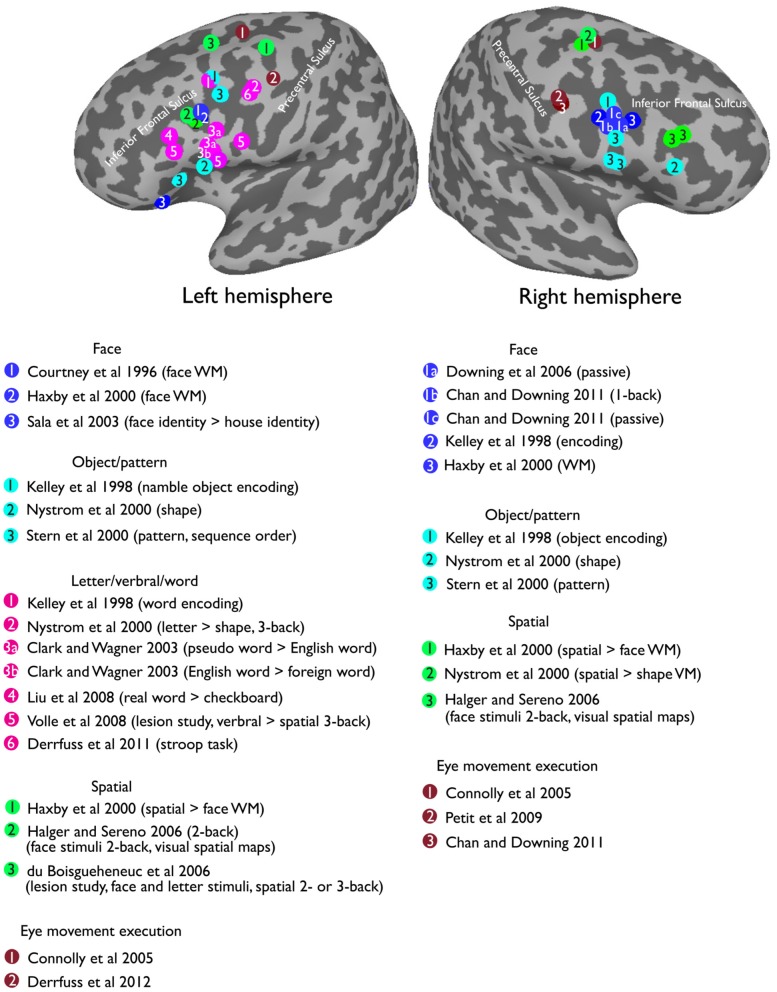
**Reported peak activations from a selection of prior studies in the lateral prefrontal cortex overlapping on a standard MNI inflated surface**.

Here, I want to argue that some seemingly inconsistent findings on the foci for face WM could result from both retinotopic and visual face biases in VLPFC. For example, it was demonstrated that the inferior frontal gyrus of the VLPFC (in the vicinity of the IFJ; Courtney et al., 1997, see Figure 5) are strongly activated during the delay period of a face WM task. However, another study (Courtney et al., 1998) found that DLPFC (along superior frontal sulcus) is specific for spatial WM, but failed to find a dissociable response in the VLPFC for face and spatial WM. Nonetheless, these different findings can be reconciled by considering the two characteristics about VLPFC, which I have mentioned earlier: (1) VLPFC has strong responses to foveal stimuli (Suzuki and Azuma, 1983; O'Scalaidhe et al., 1997; Scalaidhe et al., 1999). (2) VLPFC is selective to faces/eyes stimuli (Downing et al., 2006; Chan and Downing, 2011). Thus, the strong VLPFC activation for face WM task in Courtney et al. (1997) could be explained by the fact that their face stimuli (especially the eyes) were presented in the center of the screen. In the contrast, in their later study (Courtney et al., 1998), the lack of strong activation during their face WM task could largely be explained by the fact that face stimuli were now presented in the less-preferred peripheral position. Hence, in order to better understand the underlying factors that contribute to the functional organization of the VLPFC, it is therefore critical for researchers to also examine the effect of both visual stimuli and positional information in this region.

Recent neuropsychological studies have also provided valuable insights on objects vs. spatial cognition in the lateral prefrontal cortex. For example, patients with lesions in VLPFC and DLPFC have shown severe deficits in both object and spatial tasks (Muller et al., 2002). In addition, a recent study (du Boisgueheneuc et al., 2006) has shown that patients with lesions to the left superior frontal gyrus in the DLPFC demonstrate a deficit in spatial but not face or letter WM tasks (face and letter were centrally presented, except in spatial WM task) when compared to healthy controls. This finding suggests that DLPFC is oriented for spatial cognition and can be dissociated from object (faces and letter) processing. Lesion studies have therefore provided compelling evidence for dissociable object and spatial representations in the lateral prefrontal cortex.

So far, evidence reviewed here has pointed to the direction that the nature of responses in VLPFC appears to be a domain specific one, with direct input feeding from the visual object ventral pathway coupled with the retinotopic properties of the visual cortex and visual experience with the category. Thus, the strong right VLPFC responses for faces may be due to input from the right predominant representations for faces in the ventral pathway. With this logic, it is reasonable to consider that other categories with similar properties to faces, (categories that contain highly relevant information), elicit robust activation in ventral pathway, and a strong foveal representation should also elicit a similar response in the prefrontal cortex.

Visually presented words fit these criteria perfectly. Words are highly salient stimuli, they produce strong responses in the putative visual word form area (VWFA) in the left fusiform gyrus (Cohen et al., 2002; Baker et al., 2007; Liu et al., 2008), which also shows a robust central foveal bias (Hasson et al., 2002; Levy et al., 2004). Indeed, many studies have reported the left VLPFC involved in the processing of words or letter stimuli (Clark and Wagner, 2003; Liu et al., 2008). Further, there is some evidence suggesting an anatomical connection between the inferior prefrontal cortex and the ventral visual cortex via the inferior frontal-occipital fasciculus (IFOF). The ability to make fine grained discrimination between stimuli (e.g., faces) depends on the structural integrity of right IFOF (Thomas et al., 2008), and words or language processing is related to the left IFOF (Catani et al., 2007; Catani and Mesulam, 2008). Intriguingly, a double dissociation has been reported for word encoding in left VLPFC and picture encoding in right VLPFC (see Figure 5 for the reported peak locations from a selection of previous studies). In particular, an early study (Wagner et al., 1997) using PET reported a stronger priming effect in left VLPFC for words relative to pictures. Further, the effect is specific to left, compared with right, VLPFC. Another study (Kelley et al., 1998) supported this double dissociation by demonstrating a stronger response in the left VLFPC (Tal [–47 9 34]) during word encoding, and a stronger response in the right VLPFC (Tal [37 3 26]) during face encoding. Interestingly, equally strong bilateral activations were found when encoding pictures of namable objects (Tal [–47 7 36], [37 3 26]), suggesting that both visual and language representations were being recruited in the lateral prefrontal cortex. Furthermore, recent work has found that the left VLPFC, specifically near the left IFJ, is involved in Stroop task and task-switching paradigms (Brass and von Cramon, 2004; Brass et al., 2005; Derrfuss et al., 2005, 2012), all of which require some level of words/letter processing demand.

Taken together, the evidence suggests that the VLPFC contains visual information, which is likely reflecting connectivity from the ventral visual pathway and our visual experience. It is therefore important to further examine the VLPFC in the light of anatomical and functional connections with other regions.

## Connectivity to face, eye, and gaze responsive regions

It has been proposed (Goldman-Rakic, 1996b) that the prefrontal cortex is functionally associated with and is an extension of the ventral cortex, and that there might be strong connectivity between these cortical regions. To date, both anatomical and functional connectivity studies in non-human primates and humans have provided some evidence regarding the connection between the prefrontal and high-level visual cortex. In monkeys, connectivity between the prefrontal and high-level visual areas has been demonstrated using an injection of wheat germ agglutinin-horseradish peroxidase or fluorescent dyes (O'Scalaidhe et al., 1997). In particular, O'Scalaidhe et al. (1997) found that all of the face-selective neurons that were located in the inferior convexity received more than 95% of their input from the temporal visual cortex. These neurons received inputs from the ventral bank of the STS, as well as the neighboring inferior temporal gyrus. These regions have frequently been reported to contain face-selective neurons (Perrett et al., 1982; Peelen and Downing, 1991; Desimone et al., 1984; Perrett et al., 1985; Pinsk et al., 2005; Tsao et al., 2006; Bell et al., 2009, 2011). The connection between the inferior frontal cortex and temporal region has also been well documented by many other researchers (Kuypers et al., 1965; Jones and Powell, 1970; Ungerleider et al., 1989; Bullier et al., 1996). In particular, some (Levy and Goldman-Rakic, 2000) have argued the fact that the VLPFC receives input from the visual ventral temporal cortex, strongly support a domain specific functional organization between the VLPFC and the high-level visual cortex.

In humans, functional connectivity studies (Nummenmaa et al., 2010) have shown that near to the IFJ, activity in the middle frontal gyrus during a gaze perception task is correlated with those in the fusiform gyrus as well as the STS, suggesting that the prefrontal cortex may be recruiting other regions for gaze perception. In addition, recent advances in fiber tracking using diffusion tensor imaging (DTI) in humans has provided further insight into the connectivity between the PFC and other brain regions for face or gaze perception. In particular, the inferior front-occipital fasciculus (IFOF), which projects from the occipital-temporal cortex to the inferior and dorsal frontal lateral cortex (Thomas et al., 2008), connects the VLPFC and the ventral visual cortex. It has been demonstrated that behavioral face discrimination ability, compared to car discrimination, is correlated with the structural integrity of the IFOF, predominantly in the right hemisphere (Thomas et al., 2008).

Overall, evidence from both functional and anatomical connectivity studies along with all the evidence provide here has enhanced our insights into the nature and origin of the face or eyes representations in the VLPFC. However, it is definitely helpful for us to put all the evidence reviewed here, relating to the function, connectivity, and organization of the lateral prefrontal cortex in the context of a more general organizational principle about the brain, namely notion of domain specific vs. domain general.

## Domain specific vs. domain general

To get a better grasp on how the VLPFC is functionally organized, one could ask whether the neural response in this region is domain specific or domain general. Many have argued that the functional organization of VLPFC is domain general i.e., based on cognitive functions (Rushworth et al., 1997; Asaad et al., 1998, 2000; Duncan and Owen, 2000; Passingham et al., 2000; Rossi et al., 2007). Specifically, some studies have suggested that the VLPFC is dedicated to a series of cognitive control tasks, for example during visual associative learning (such as visual matching task, Passingham et al., 2000; or stimulus selection, Rushworth et al., 1997), manipulation of information during WM task (Wager and Smith, 2003; Owen et al., 2005), or resolving response conflicts (e.g., Stroop task, task switching paradigm; Brass and von Cramon, 2004; Brass et al., 2005). In particular, Derrfuss et al., 2005 seems to provide some evidence to support the domain general account by demonstrating that the VLPFC elicited activations during multiple tasks (Stroop task, verbal n-back task, and task-switching paradigm). However, as mentioned before, some of the activations may be primarily driven by the stimuli rather than the tasks per second. Others have also suggested VLPFC is involved in attentional control for coordinating complex behavior. For example, previous studies have suggested that the lateral prefrontal cortex might contain neural mechanism for selective attention (Kastner et al., 1999; Corbetta et al., 2000; Hopfinger et al., 2000; Rossi et al., 2007, 2009). In particular, using a target detection task (Hopfinger et al., 2000), the VLPFC was found to show robust activation for the central cues but not the targets (checker board stimuli presented in the peripheral), claiming that the region is involved in attentional control, and thereby is critical for planning for action.

In sum, under the domain general view, sensory information is integrated in the lateral prefrontal cortex so that humans can perform a diverse range of tasks, and this cortex requires vast connections to other cortical regions processing visuospatial and motor modalities. Such cortical regions include motor cortex, FEF, posterior parietal cortex, high-level visual cortex, as well as other subcortical structures (e.g., limbic structures) (Miller, 2000; Rossi et al., 2007). Furthermore, recent theories have suggested that instead of multiple sub-regions within the lateral prefrontal cortex specializing in each of these different cognitive processes; neurons in the prefrontal cortex are believed to be highly flexible and are adaptive to support a diverse range of goal-directed behavior (Duncan, 2001; Miller, 2000).

However, as discussed earlier, others have claimed that responses in the lateral prefrontal cortex are domain (content) specific; the responses are mainly driven by different inputs from the ventral visual cortex or dorsal parietal cortex (Wilson et al., 1993; Kelley et al., 1998; Scalaidhe et al., 1999; Goldman-Rakic, 2000). In this respect, the organization of the human lateral prefrontal cortex has been largely divided into two components—object vs. spatial WM (McCarthy et al., 1996; Owen et al., 1996; Courtney et al., 1997, 1998; Haxby et al., 2000b). In the context of face, eye, and word responses in the VLFPC, evidence reviewed here seems to further support the domain specific view, where the stronger responses are specific for the preferred stimuli, and these preferred responses also reflect the retinotopic position represented in the high-level visual cortex (Rainer et al., 1998; Hagler and Sereno, 2006; Chan et al., 2010; Kravitz et al., 2010; Chan and Baker, 2011; Voytek et al., 2012), which is likely to be fed forward by the strong anatomical connections between the occipito-temporal cortex and inferior prefrontal cortex. Hence, both visual category and positional information can be present in the VLPFC.

At the core of this debate lies the question of how the VLPFC maintains long-term stable representations while concurrently processing a vast amount of information flexibly and efficiently (O'Reilly, 2010). Indeed, to achieve stability, the VLPFC has to contain some degree of functional specialization to tie to other more posterior cortical regions, reflected in part through separate inputs from the dorsal and ventral visual pathways (O'Reilly, 2010; Kravitz et al., 2013). The strong connections, both anatomical and functional, between the VLPFC and high-level visual cortex may provide a basic content-specific processing framework that underlies VLPFC function. Finally, it remains possible that multiple organizational principles (beyond the domain general vs. domain specific distinction) are reflected in the shared “activation maps” frequently seen across different studies (see Op de Beeck et al., 2008, for a similar discussion in the context of high-level visual cortex). The strong responses elicited with specific paradigms may simply be driven by a single optimal stimulus or condition for any of these organizational principles. Taking a broad perspective across multiple studies may provide clues regarding the overall functional organization of the lateral prefrontal cortex.

## Conclusions and future directions

This paper has provided a summary account for understanding the nature and origin of category specific responses in the VLFPC. The robust responses for faces, eyes, and words in the VLPFC (overlaps with the IFJ) could be due to eccentricity biases fed forward from the ventral visual cortex, which is likely to reflect our visual experience and statistics of the visual world. The strong activation in the right IFJ for eyes in particular (Chan and Downing, 2011) is probably one of the special cases; the activation is largely driven by both category and foveal biases in VLPFC, which is inherited from the strong connectivity to other regions in the extended face network (Haxby et al., 2000a; Ishai et al., 2005; Avidan and Behrmann, 2009). In addition, the strong responses for faces in the absence of WM in the IFJ may well be reflecting the “steady-state” properties of the prefrontal cortex for highly salient and evolutionary relevant stimuli. Future work will need to address the functional and anatomical connectivity between IFJ and other face or eyes responses regions such as STS, amygdala, as well as regions that involved in gaze execution (Connolly et al., 2005; Petit et al., 2009) such as FEF and supplementary eye fields.

In order to further understand the underlying organizational principles of the lateral prefrontal frontal cortex, future work will also need to examine the functional connectivity between the prefrontal and the ventral visual cortex by taking advantage of the fact that some categories that require fine-grain visual analysis (such as faces and words) have strong foveal representations in the ventral visual cortex, whereas other categories that require coarser visual analysis (such as tools and scenes) have strong peripheral representations in the visual cortex. There is also a need to carefully map out the response profiles in the VLPFC using a wide range of visual categories that shows different retinotopic biases in VLPFC and ventral visual cortex. Furthermore, to test the effect of visual experience on the VLPFC, and whether the VLPFC is malleable to learning and experience, future investigation should also focus on manipulating the level of visual experience or degrees of visual analysis using novel objects. In addition, transcranial magnetic stimulation (TMS) can be employed to disrupt activity in the face selective occipital area (Yovel and Kanwisher, 2005; Pitcher et al., 2011). This may potentially allow us to establish the connectivity between face selective visual region in the ventral cortex with the face selective frontal area IFJ. This line of research will definitely enhance our insight into the development and evolution of the lateral prefrontal cortex.

### Conflict of interest statement

The author declares that the research was conducted in the absence of any commercial or financial relationships that could be construed as a potential conflict of interest.

## Key Concept

Working memory: Refers to the ability that humans can maintain and manipulate multiple types of information online; information can be visual, spatial, verbal or tactile. It is thought that the prefrontal cortex is critical in manipulating information in working memory.
Domain general: Refers to the theoretical view that information is organized in terms of a wide range of cognitive process, context, or tasks.
Domain specificity: Refers to the theoretical view that information is organized based on a particular type of information or content.
The ventral pathway: Is a visual pathway originates from the occipital cortex and extends into the temporal cortex. This pathway is also characterized as the “what” pathway, and is thought to be important for object recognition.
The dorsal pathway: Is a visual pathway originates from the occipital cortex and extends into the parietal cortex. The dorsal pathway is also characterized as the how or where pathway, and is thought to be critical for processing of spatial information.
